# Small networks of expressed genes in the whole blood and relationships to profiles in circulating metabolites provide insights in inter-individual variability of feed efficiency in growing pigs

**DOI:** 10.1186/s12864-023-09751-1

**Published:** 2023-10-27

**Authors:** Camille Juigné, Emmanuelle Becker, Florence Gondret

**Affiliations:** 1 PEGASE, INRAE, Institut Agro, Saint-Gilles, F-35590 France; 2 University Rennes, Inria, CNRS, IRISA - UMR 6074, Rennes, F-35000 France

**Keywords:** Feed efficiency, Fatty acids, Metabolomic, Molecular modules

## Abstract

**Background:**

Feed efficiency is a research priority to support a sustainable meat production. It is recognized as a complex trait that integrates multiple biological pathways orchestrated in and by various tissues. This study aims to determine networks between biological entities to explain inter-individual variation of feed efficiency in growing pigs.

**Results:**

The feed conversion ratio (FCR), a measure of feed efficiency, and its two component traits, average daily gain and average daily feed intake, were obtained from 47 growing pigs from a divergent selection for residual feed intake and fed high-starch or high-fat high-fiber diets during 58 days. Datasets of transcriptomics (60 k porcine microarray) in the whole blood and metabolomics (1H-NMR analysis and target gas chromatography) in plasma were available for all pigs at the end of the trial. A weighted gene co-expression network was built from the transcriptomics dataset, resulting in 33 modules of co-expressed molecular probes. The eigengenes of eight of these modules were significantly ($P \le 0.05$) or tended to be ($0.05 < P \le 0.10$) correlated to FCR. Great homogeneity in the enriched biological pathways was observed in these modules, suggesting co-expressed and co-regulated constitutive genes. They were mainly enriched in genes participating to immune and defense-related processes, and to a lesser extent, to translation, cell development or learning. They were also generally associated with growth rate and percentage of lean mass. In the whole network, only one module composed of genes participating to the response to substances, was significantly associated with daily feed intake and body adiposity. The plasma profiles in circulating metabolites and in fatty acids were summarized by weighted linear combinations using a dimensionality reduction method. Close association was thus found between a module composed of co-expressed genes participating to T cell receptor signaling and cell development process in the whole blood and related to FCR, and the circulating concentrations of polyunsaturated fatty acids in plasma.

**Conclusion:**

These systemic approaches have highlighted networks of entities driving key biological processes involved in the phenotypic difference in feed efficiency between animals. Connecting transcriptomics and metabolic levels together had some additional benefits.

**Supplementary Information:**

The online version contains supplementary material available at 10.1186/s12864-023-09751-1.

## Introduction

In the context of various geopolitical tensions and societal questions on the agri-agro-food systems, feed efficiency (FE) is a research priority to support food security and a sustainable meat production. Indeed, better FE is associated with a reduced amount of feeds needed for production and lower environmental wastes and emissions. Feed efficiency is measured on farms by the feed conversion ratio (FCR), an index calculated as the ratio of feed intake to body weight (BW) gain during a test period. Residual feed intake (RFI) has been also proposed as a refined measure of FE for genetic selection; it is calculated as the difference between observed feed intake and predicted feed intake from production and maintenance needs, which allows RFI to reflect digestive and metabolic variabilities [1, 2]. A number of studies have been performed in the past years to depict and understand the biological bases of FE. Based on the comparison of animals with low or high FCR or RFI, they all concluded to the complex nature of FE since this trait integrates multiple biological pathways orchestrated in and by various tissues [2–4]. Among tissues, peripheral blood is a convenient and relatively easy sampling source of biological information that can highlight the variations in tissues metabolism and physiology to understand complex phenotypes [5] and with potential outcomes for applications in diagnostics and selection [6]. In pigs, about one thousand genes were found differentially expressed in the whole blood between two lines of pigs divergently selected for RFI [7, 8]. Gene set enrichment analysis on the whole blood transcriptome in beef cattle has also allowed identifying biological pathways associated with a divergent selection for low or high RFI [5]. Moreover, we recently showed that gene expression profiling in the whole blood is suitable to identify a few number of molecular candidate biomarkers for FCR in growing pigs, when gene expression levels were analyzed by machine learning algorithms based on classification or regression trees [9]. Likewise, metabolomic studies have shown that circulating concentrations of metabolites in the blood can be related to economically-important traits including FE [10]. However, all these approaches did not address the inter-individual variation in FE traits and did not attend to depict the interactions among the biological entities at a given level or different levels of omics organization. Therefore, a systemic approach considering the multiple relationships between molecules can add new insights in the architecture of complex traits such as FE.

Among various systems biology approaches, the weighted gene co-expression network analysis (WGCNA) has been proposed as a suitable method for defining interactions between transcripts of genes, by grouping them in modules of pairwise correlations to reveal the higher-order organization of the transcriptome [11]. Based on RNA-sequencing data in the liver, two co-expression networks were identified as associated with high or low FE in dairy cows [12]. Another approach based on linear models allowed to combine gene expression data and high throughput metabolomics data in skeletal muscle [13]; in this study, pairs of metabolite-transcript associated with sphingolipid catabolism, multicellular organismal process, and purine metabolic processes were associated with differences in FE between two pig breeds and between two groups of pigs of low or high FE values.

The aim of the present study was to depict the biological bases underlying inter-individual variability in FE and other related traits in growing pigs by identifying small networks of interconnected gene transcripts in the whole blood and their relationships with global profiles of circulating metabolites. Eight molecular modules mainly composed of interconnected genes involved in immune and defense-related processes, were related to variability between pigs in FCR. Other important biological processes represented in these gene networks were the response to organic substance, ribosome biogenesis and translation, and cell development and learning, respectively. One module of inter-connected expressed genes related to immune process was associated with circulating concentrations of omega3 fatty acids in the plasma, thus connecting transcripts to metabolites in the determinism of variability in FE.

## Material and methods

### Ethics

We reused transcriptomics and metabolomics datasets acquired in the whole blood from purebred French Large White pigs produced in a divergent selection experiment for RFI [7, 14], and that have been previously analyzed separately and for the line-associated effects only. The animal phenotypes have been published by [15]. These data were complemented by data on circulating concentrations of fatty acids (FA) in the plasma of the same pigs to specify lipid-related processes, that have not been previously published. In the original publications, the care and use of pigs were performed in compliance with the European Union legislation (directive 2010/63/EU). The protocol was approved by an Ethics Committee in Animal Experiment (Comité Rennais d’Ethique en matière d’Expérimentation Animale, CREEA N${}^{\circ }$007, agreement N${}^{\circ }$07-2012). All animals were reared and killed in compliance with the national regulations and according to procedures approved by the French veterinary Services. All methods were reported in accordance with ARRIVE guidelines. In the present study, reusing published data to perform new analyses for different animal traits perfectly fits with the 3R (Replacement, Reduction and Refinement) principles.

### Origin of phenotypic data

Full description of the experimental design that provided the original datasets is referenced by [15]. Briefly, data were obtained from a total of 48 growing pigs (barrows) of two lines in the 7th generation of divergent selection for RFI, and fed diets formulated at isocaloric and isoproteic bases but differing in energy source and nutrients (lipids and fibers vs. starch), were tested from $74 d \pm 0.3 d$ of age to $132.5 d \pm 0.5 d$ (SEM) of age. All pigs were reared in isolated pens during the test period to allow the control of spontaneous feed intake, thus minimizing also the usual pen effect when pigs are reared in groups. From this publication, we considered body weight at slaughter (BW in kg), age at slaughter (in days), average daily feed intake (ADFI, in g/day), average daily gain (ADG, in g/day), FCR (calculated as the ratio between ADFI and ADG during the feeding trial), and the percentage (relative to carcass weight) of the dorsal subcutaneous adipose tissue (%backfat) and of the loin muscle cut (%loin) as surrogates of body composition.

### Transcriptomic dataset

The transcriptomic data were retrieved from NCBI’s Gene Expression Omnibus (GEO) Subserie accession number GSE70838 (http://www.ncbi.nlm.nih.gov/geo/query/acc.cgi?acc=GSE70838). These data have been obtained from the whole blood collected in pigs at $132.5 d \pm 0.5 d$ (SEM) of age, by using a custom porcine microarray ($8\times 60K$, GPL16524, Agilent Technologies France, Massy, France) containing 60, 306 porcine probes. Full description of the methods to produce the raw microarray data can be found in [7]. After quality filtration based on four criteria (background intensity value, diameter, saturation and uniformity of the spot), the original dataset contained 26, 322 annotated probes expressed in the whole blood. There were approximately 2.2 replicates per unique gene in the transcriptomic dataset (min: 1; max: 33).

### Metabolomic dataset

The metabolomic dataset consisted in high resolution 1H-NMR spectra generated in plasma of the 48 pigs, and was retrieved from Jegou et al. [14]. The generated spectra were processed with one level of zero-filling and Fourier transformation after multiplying free induction decays by an exponential line broadening function of 0.3 Hz. The 1D NMR spectra were manually phase- and baseline-corrected, and referenced to the chemical shift of the alpha-glucose at delta 5.235. The bin area method was used to segment the spectra between 0.6 and 8.5 ppm using the intelligent variable size bucketing tool. Bin areas generated a matrix, which was normalized by dividing each integrated segment by the total area of the spectrum. This resulted in a new matrix that is used to perform statistical analyses. A total of 94 buckets, and consequently of 94 variables in this matrix, was considered for dedicated analyses in the present study. Buckets are individual metabolites or groups of two metabolites. They were notably assigned to different amino acids, creatine, lactate and glucose.

When using the 1H-NMR approach, lipids were all grouped as a single spectrum. In the present study, to investigate more deeply the role of the lipidic family in FE variability, the circulating concentrations of fatty acids (FA) in plasma were newly determined in the 48 pigs. For that, lipids were extracted from plasma as previously described for tissues [15], and methylated. Dedicated analyses were then performed on a gas chromatograph (Nelson Analytical, Manchester, NH) equipped with a fused-silica capillary column ($30 m\ \times \ 0.25 mm$ internal diameter), with a base-deactivated silica stationary phase (a $0.25-\mu m$ film thickness) filled with a stationary phase (80% biscyanopropyl and 20% cyanopropylphenyl) and using margaric acid (C17:0) as the internal standard. The furnace temperature was $180^{\circ }C$, and injector and detector temperatures were $240^{\circ }C$. Retention times and peak areas were determined. Peaks were identified by comparison with the retention times of standard FA methyl esters. Individual FAs were then quantified as percentages of the sum of FA identified in each sample. To facilitate the biological interpretation, FA have been then grouped into families of saturated FA with 14C or less (sC14:0), polyunsaturated FA of the omega-6 family (ssn-6c), and polyunsaturated FA of the omega-3 family (ssn-3). All other identified FA were kept as these. This led to a total of 14 variables representing circulating FA concentrations in plasma (Supplementary Table 1).

### Construction of the weighted gene co-expression networks

Starting from a matrix whose individuals are pigs and features are probes expression levels (48 pigs $\times$ 26, 322 probes), we performed a hierarchical clustering to identify outlier individuals as recommended [16]. One pig was detected as an outlier and further removed from the dataset due to aberrant values. Then, we quantified the number of probes that were significantly linked to the animal phenotypic traits of interest (linear models with a *P*-$value < 0.05$ as cut-off). The results of the linear regressions showed that age at slaughter and line were the factors affecting the most the expression levels of molecular probes (19% of the probes significantly affected by age at slaughter and 14% by the line effect) (Supplementary Table 2). For next steps of analysis, we used the residuals of these linear regressions for the age effect, but preserved the intra- and inter-line variability of FE.

Corrected probe expression levels were then filtered. Only probes *i* whose log fold-change $FC_i$ was greater than 1 were selected, with

1 $$\begin{aligned} log_2(FC_i)\ =\ max(log_2(expression_i)) - min(log_2(expression_i)) \end{aligned}$$

Indeed, we considered that the variant probes formed a reduced dataset in which connected probes can be more easily found when reducing noise and spurious associations. A smaller dataset is also associated with a less computationally demanding analysis for analyzing gene networks. This reduced dataset included 16, 190 probes for the 47 pigs.

To calculate the co-expression network, we used the Weighted Gene Co-expression Network Analysis (WGCNA) step-by-step method [11], performed with R 4.2.2. The first step consisted in calculating a measure of co-expression similarity $s_{i,j}$ between each pair of probes to highlight the pairs of probes whose expression varies in a similar way. The adjacency matrix $A = [a_{i,j}]$ was then constructed by raising the co-expression similarity measure $s_{i,j}$ to the power $\beta$, using the signed hybrid method (only positive correlations were kept) [Eq. 2].

2 $$\begin{aligned} signed\ hybrid\ a_{ij} = \left\{ \begin{array}{ll} {cor(x_i,x_j)}^{\beta } &{} cor(x_i,x_j)>0 \\ 0 &{} cor(x_i,x_j)<0 \end{array}\right. \end{aligned}$$

where $a_{ij}$ is the element (*i*, *j*) of the adjacency matrix *A*, $\beta$ is the soft-thresholding, $x_i$ is the level expression of the *i*th probe, and $cor(x_i,x_j)$ is the Pearson correlation between expression profiles of the *i* and *j* probes.

$\beta$ is a non-dichotomic soft thresholding that allows to evaluate connection between probes without losing the continuous character of the co-expression. Low correlations are better masked with high $\beta$ values. We set $\beta$ to 6 according to the criterion of the “approximate scale free topology” [17].

Considering probes as nodes, a weighted co-expression network can be then deduced from the adjacency matrix, by adding edges of weight $a_{ij}$ between pairs of probes whose weight is strictly positive.

### Detection of modules of co-expressed probes and their relationships with animal phenotypic traits

To detect modules in this network of co-expressed probes, a proximity measure was calculated using the Topological Overlap Measure [18], which is a valuable similarity measure set as default approach in WGCNA framework and a hierarchical clustering was performed using the standard R function “clust” and the “average” agglomeration method. This results in a dendrogram, in which clusters can be detected using the dynamicTreeCut algorithm [11] and a sensitivity threshold set to 2. To keep modules of highly co-expressed genes, we set the minimum module size to 25 probes. Each module was then summarized by its first principal component called the module eigengene (ME), that is a mathematical solution to condense the expression profile of the probes in the module.

A heatmap of correlations between ME and phenotypic traits was then produced. The heatmap can be examined to find the most significant associations. In this study, we considered $P\le 0.05$ as significant association and $0.05 < P \le 0.10$ as a tendency.

We also calculated the gene significance (GS) as (the absolute value of) the correlation between the probe and the phenotypic trait. For each module, we defined the quantitative measure of module membership (MM) as the correlation of ME and the gene expression profile. Using both the GS and MM measures, we can identify genes that have a high significance for FE traits (central players) and high module memberships in the modules. For a subset of modules, we provide a graphical representation of the sub-networks considering the annotated probes only. For that, pairs of probes with correlation coefficients greater than the 95th percentile in a given module were selected from the adjacency matrix, and represented using Cytoscape with nodes corresponding to probes and edges corresponding to the adjacency matrix values. For probes with low correlation with other probes, edges were represented in a different color.

### Biological functional enrichment in modules of co-expressed probes

For modules that were significantly related to FCR based on the heatmap examination between module eigengenes (ME) and animal traits, we matched their constitutive molecular probes to the corresponding unique genes (official gene symbol), using the annotation provided by the manufacturer of the expression microarray. When applicable, the gene ontology (GO) terms for biological processes were then automatically searched in each module, using the Database for Annotation, Visualization and Integrated Discovery (DAVID) bioinformatics knowledgebase v2022q4 released (http://david.abcc.ncifcrf.gov/). The GOBP terms_FAT were selected to filter the broadest terms. The results were downloaded using the “Functional annotation clustering” option of the DAVID tool. Only clusters of terms with an enrichment score (measured by the geometric mean of the EASE score of all enriched annotations terms) greater than 1.2 were considered. Within each cluster, the top GO term was listed together with its own enrichment score and the associated modified Fisher exact *P*-*value*.

### Establishing profiles of circulating metabolites and evaluating connections between metabolic and transcriptomic levels

The second and third datasets considered in this study encompassed the circulating concentrations of metabolites and of FA, respectively. To reduce the dimension and facilitate correlation analyses with gene expression networks, these datasets were each summarized by Principal Component Analysis (PCA) using the R packages FactoMineR and factoextra [19, 20]. This would avoid bias when considering hundreds metabolic variables with only few modules of highly correlated genes. A PCA was used to summarize the profile in metabolites identified after 1H-NMR analysis (94 variables) and another PCA was used for circulating concentrations of FA (14 variables). From each table kept separately (one for 1H-NMR analysis and one for FA), PCA transforms the original (mean-centered) observations to a new set of variables (dimensions) using the eigenvectors and eigenvalues calculated from a covariance matrix of the original variables. The first components of the PCA were called Dim*i*_Metab for the metabolomic 1H-NMR table and Dim*i*_FA for the FA table, respectively, with *i* = 1 to 5. These dimensions were linear combinations of the original variables.

To connect information at the two omics levels, the dimensions of each PCA were then correlated to the eigengenes of the WGCNA modules (ME) by using Pearson correlations. The correlations were represented by heatmaps to facilitate the description.

## Results

Data obtained in a total of 47 growing pigs with inter-individual differences in FCR (i.e., the measure of FE on farms) due to genetic selection for RFI and to the diet received during a test period of 58 days, were considered in the present study. In addition to FCR, the average daily gain (ADG) and average daily feed intake (ADFI) (i.e., the two components of FCR), and body composition estimated by percentage (relative to carcass weight) of backfat (%backfat) and of the loin cut (%loin) were also obtained (Supplementary Table 1).

### Definition of gene co-expression network in the whole blood of pigs

A network was built with the WGCNA package from the expression levels of 16, 190 molecular probes expressed in the whole blood, where nodes correspond to the expression profile of the molecular probes, and edges are determined by the pairwise correlations between probes expression (the adjacency matrix is available at https://data-access.cesgo.org/index.php/s/YPz0J2ItxIEuN5M). This network was then analysed to find modules defined as groups of co-expressed probes that may represent the molecular architecture behind the animal phenotypic traits.

**Fig. 1:**
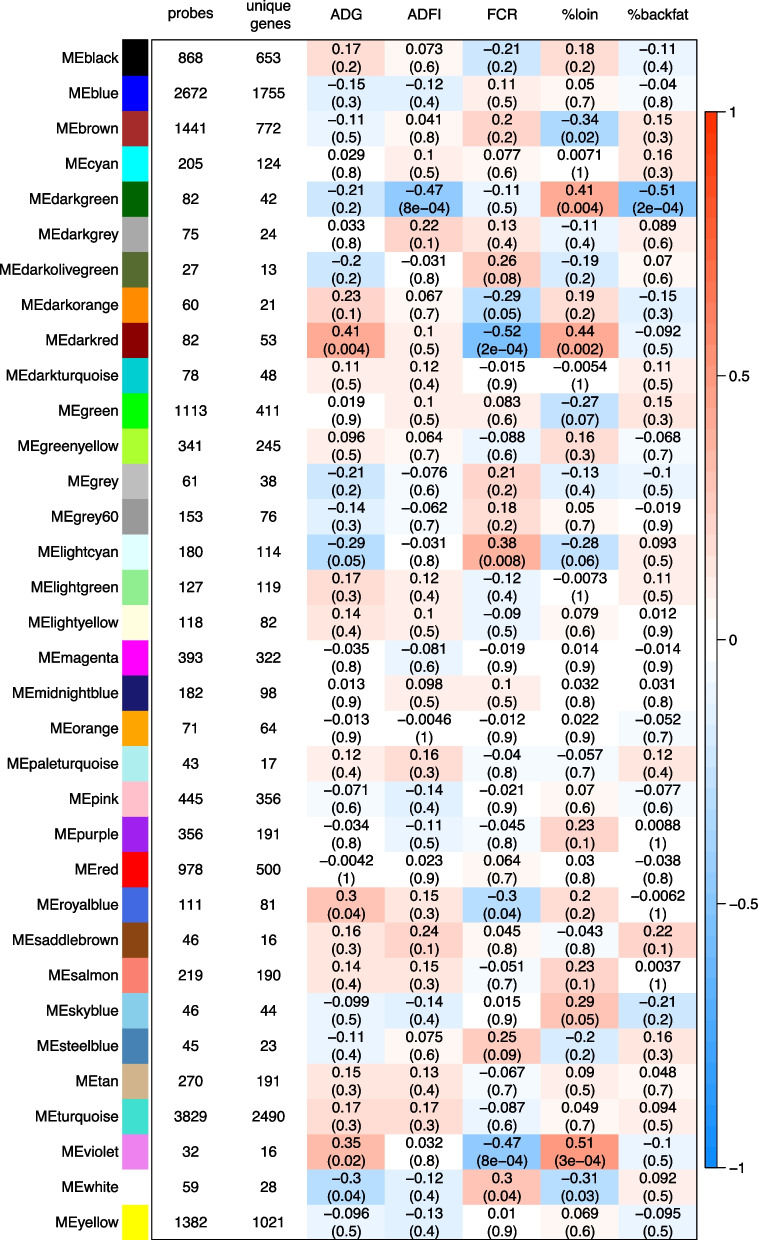
Heatmap of correlations between module eigengenes and animal phenotypic traits. Module eigengene (ME) was the representative of gene expression profile in the module of co-expressed molecular probes elicited from the weighted gene correlated network analysis (WGCNA) from microarray data in the whole blood of 47 growing pigs. Animal phenotypic traits were recorded during a test period of 58 days. The heatmap indicates the Pearson correlation coefficient between ME and the phenotypic trait together with the statistical significance (*Pvalue*). Abbreviations: ADG = average daily gain; ADFI = average daily feed intake; FCR = feed conversion ratio; %loin = percentage of loin weight relative to carcass weight; %backfat = percentage of dorsal subcutaneous fat tissue weight relative to carcass weight; ME = module eigengene

After excluding the grey module which is used to hold all the probes that do not clearly belong to any other modules, we show that the co-expression network was composed of 33 modules composed of 27 to 3, 829 molecular probes (Supplementary Table 3). Annotations were used when applicable to identify the corresponding genes. The distribution in the number of probes and their corresponding unique genes per module is shown in Fig. 1.

The module eigengene (ME) was then calculated as the representative of expression profiles of the genes in the module. The module membership (MM) was calculated by correlating ME and the expression profile of the probes within each module. By definition, the closer to 1 or $-1$ is MM, the higher is the gene connected to ME. The medians of the MM values indicated a satisfactory clustering from the whole network of the molecular probes expressed in the whole blood of the 47 pigs. The values are available in Supplementary Table 3.

### Relationships between modules of co-expressed genes and animal phenotypic traits

The heatmap of the correlation coefficients between the module eigengene (ME) of each WGCNA module and the animal phenotypic traits is presented in Fig. 1. For six modules, the ME was significantly correlated (*P*-$value\le 0.05$) with FCR: they were the modules violet (probes: 32, unique genes: 16, *P*-$value = 8e-04$), darkred (probes: 82, unique genes: 53, *P*-$value = 2e-04$), royalblue (probes: 111, unique genes: 81, *P*-$value = 0.04$), lightcyan (probes: 180, unique genes: 114, *P*-$value = 0.008$), white (probes: 59, unique genes: 28, *P*-$value = 0.04$), and darkorange (probes: 60, unique genes: 21, *P*-$value = 0.05$). For two other modules, ME tended (*P*-$value < 0.1$) to be correlated with FCR: they were the modules darkolivegreen (probes: 27, unique genes: 13, *P*-$value = 0.08$) and steelblue (probes: 45, unique genes: 23, *P*-$value = 0.09$).

For the modules violet, darkred, royalblue, lightcyan, and white, the correlations between ME and ADG were also significant, and for the module darkorange, there was a trend for correlation between ME and ADG. As expected, the signs of correlation between ME and ADG and between ME and FCR (which is the ratio between ADFI and ADG during the test period), were opposite. For the modules darkolivegreen and steelblue, the correlation coefficients between ME and ADG did not reach statistical significance.

Four of the eight modules associated with FCR also displayed significant correlations with %loin (with opposite signs of correlation): the modules violet, darkred, lightcyan and white. For these modules, there was no significant correlation between ME and %backfat.

Finally, none of the eight modules associated with FCR were significantly related to ADFI. In the whole network, only the darkgreen module (probes: 82, unique genes: 43, *P*-$value = 8e-04$) was highly correlated with ADFI; it was also significantly related with %loin (*P*-$value = 0.004$) and %backfat (*P*-$value = 2e-04$). The saddlebrown module (probes: 46, unique genes: 12) tended to be correlated with ADFI (*P*-$value = 0.1$) and with %backfat (*P*-$value = 0.1$). Finally, the eigengenes of the modules green (probes: 1, 113, unique genes: 412, *P*-$value = 0.07$), brown (probes: 1, 441, unique genes: 773, *P*-$value = 0.02$) and skyblue (probes: 46, unique genes: 45, *P*-$value = 0.05$) displayed significant associations with %loin, but without significant correlations with any other animal phenotypic traits.

### Close-vicinity of the different modules of co-expressed genes

**Fig. 2:**
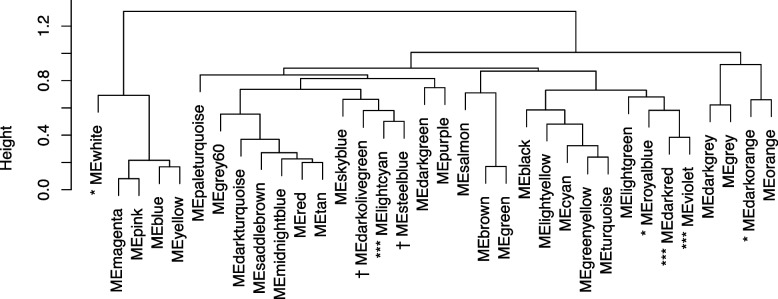
Hierarchical clustering of module eigengenes. The modules that were found highly correlated with feed conversion ratio (FCR) are enlightened (*** $P \le 0.001$, * $P \le 0.05$, and $\dagger$ $0.05 < P \le 0.10$)

To evaluate the connectivity between modules in the network, a hierarchical clustering was performed between the eigengenes (ME) of the modules. The resulting dendrogram is shown in Fig. 2, in which the eight modules that were significantly associated or tending to be associated with FCR are enlightened. Among these eight modules, two clusters were identified. The first cluster associated the lightcyan (114 genes), steelblue (23 genes) and darkolivegreen (13 genes) modules. The second cluster associated the darkred (53 genes), violet (16 genes) and royalblue (81 genes) modules. The white (28 genes) and the darkorange (21 genes) modules were isolated in the dendrogram. In addition, the green (411 genes) and brown (772 genes) modules, that were significantly associated with %loin, were clustered together (Fig. 2).

### Functional enrichment of the modules in biological processes

For the eight modules identified as significantly correlated or tending to be correlated with FCR, an enrichment analysis was performed to find the main biological processes shared by the co-expressed gene transcripts within each module (Table 1). Annotations of the probes were first retrieved, and the corresponding gene name was associated to each probe when applicable. The DAVID tool was used on the gene list uploaded for each module.

**Table 1:** Functional enrichment in biological pathways for molecular modules related to FCR

Module	probes	unique genes	match ID DAVID	GO_terms
darkolivegreen	27	13	9	GO:0006954 inflammatory response $E=15.6\ P <0.05$
darkorange	60	21	15	GO:0010033 response to organic substance $E=4.4\ P<0.001$,
				GO:0002376 immune system process $E=2.9\ P<0.05$
darkred	82	53	44	GO:0042273 ribosomal large subunit biogenesis $E=18.8\ P <0.01$,
				GO:0002181 cytoplasmic translation $E=16.5\ P < 0.01$
lightcyan	180	114	96	GO:0007166 cell surface receptor signaling pathway $E=2.3\ P <0.001$,
				GO:0032502 developmental process $E=1.5\ P <0.001$,
				GO:0006955 immune response $E=2.74\ P < 0.001$,
				GO:0006909 phagocytosis $E=4.5\ P <0.01$,
				GO:0042113 B cell activation $E=9.8\ P<0.001$,
				GO:0070887 cellular response to chemical stimulus $E=1.7\ P<0.01$,
				GO:0045088 regulation of innate immune response $E=3.4\ P<0.05$,
				GO:0050727 regulation of inflammatory response $E=3.1\ P<0.05$
royalblue	111	81	71	GO:0050852 T cell receptor signaling pathway $E=21.1\ P <0.001$,
				GO:0030155 regulation of cell adhesion $E=5.6\ P<0.001$,
				GO:0048869 cellular developmental process $E=2.0\ P<0.001$
steelblue	45	23	10	GO:0008104 protein localization $E=3.9\ P <0.05$
violet	32	16	12	GO 0045087 innate immune response $E = 9.4\ P<0.001$,
				GO:0016567 protein ubiquitination $E = 7.5\ P<0.05$,
				GO:0006952 defense response $E = 6.0\ P<0.001$
white	59	28	23	GO:0072359 circulatory system development $E=4.4\ P < 0.01$,
				GO:0007612 learning $E=16.9$ $P < 0.01$,
				GO:0030036 actin cytoskeleton organization $E=5.0\ P < 0.05$

The DAVID tool was used to identify the top enriched pathways across the list of unique annotated genes within each module. The gene ontology (GO) terms for biological processes are indicated together with enrichment score (E) of the process and Fisher *P* value

The lightcyan which was the biggest module correlated to FCR (180 probes corresponding to 114 unique genes), displayed a large number of different clusters of biological processes. On the opposite, for the other seven modules considered, there was/were only one to three clusters ($E > 1$) identified among the GO terms in each module (Table 1). This indicates a good consistency of the biological processes shared by the intra-connected genes within each module. The modules violet, royalblue, darkorange, lightcyan and darkolivegreen showed a predominance of immune, inflammatory and defense-related pathways across their constitutive genes. The darkorange module also included genes involved in the response to organic substance. The darkred module was rather oriented towards ribosome biogenesis and the process regulating translation. The white module was related to circulatory cell development and to learning.

The biological processes identified in the other modules related to feed intake (ADFI) or body composition (%loin and %backfat), but not to FCR, were described in Table 2.

**Table 2:** Functional enrichment of molecular modules correlated with feed intake or body composition, but not to FCR

Module	probes	unique genes	match ID DAVID	GO_terms
brown	1,441	772	623	GO:0009966 regulation of signal transduction $E=1.4\ P < 0.001$,
				GO:0050790 regulation of catalytic activity $E=1.4\ P < 0.001$,
				GO:0007169 transmembrane receptor protein tyrosine kinase signaling pathway $E=1.9\ P < 0.001$,
				GO:0036211 protein modification process $E=1.3\ P < 0.001$,
				GO:0042886 amide transport $E=1.4\ P < 0.001$,
				GO:0007167 enzyme linked receptor protein signaling pathway $E=1.7\ P < 0.001$,
				GO:0031400 negative regulation of protein modification process $E=1.9\ P < 0.001$,
				GO:0042692 muscle cell differentiation $E=1.8\ P < 0.01$,
				GO:0071363 cellular response to growth factor stimulus $E=1.9\ P < 0.001$,
				GO:0031331 positive regulation of cellular catabolic process $E=1.7\ P < 0.05$,
				GO:0008654 phospholipid biosynthetic process $E=2.5\ P < 0.01$,
				GO:1903320 regulation of protein modification by small protein conjugation or removal $E=2.2\ P < 0.01$,
				GO:0045595 regulation of cell differentiation $E=1.4$ $P < 0.01$,
				GO:0034284 response to monosaccharide $E=2.1\ P < 0.01$
darkgreen	82	42	33	GO:0009636 response to toxic substance $E=9.9\ P < 0.01$,
				GO:0032496 response to lipopolysaccharide $E=7.9\ P < 0.01$,
				GO:0051128 regulation of cellular component organization $E=2.2$ $P < 0.05$,
				GO:0042063 gliogenesis $E=8.9\ P < 0.01$
green	1,113	411	356	GO:0006325 chromatin organization $E=2.0\ P < 0.001$,
				GO:0009891 positive regulation of biosynthetic process $E=1.7\ P < 0.001$,
				GO:0034968 histone lysine methylation $E=5.3\ P <0.001$,
				GO:0033555 multicellular organismal response to stress $E=4.8\ P <0.01$,
				GO:1901699 cellular response to nitrogen compound $E=2.1\ P <0.001$,
				GO:0032922 circadian regulation of gene expression $E=5.7\ P <0.01$,
				GO:0006622 protein targeting to lysosome $E=9.37$ $P <0.001$,
				GO:0016050 vesicle organization $E=2.4$ $P <0.001$,
				GO:0071407 cellular response to organic cyclic compound $E=2.0\ P <0.01$,
				GO:0016071 mRNA metabolic process $E=2.0\ P <0.001$,
				GO:0009611 response to wounding $E=2.1$ $P <0.01$
saddlebrown	46	16	13	GO:0006357 regulation of transcription $E=3.72\ P <0.01$,
				GO:0009628 response to abiotic stimulus $E=9.6$ $P <0.001$
skyblue	46	44	36	GO:0006807 nitrogen compound metabolic process $E=1.80\ P < 0.001$,
				GO:0045184 establishment of protein localization $E=2.7\ P < 0.01$,
				GO:0034660 ncRNA metabolic process $E=4.8\ P <0.05$

The DAVID tool was used to identify the top enriched pathways across the list of unique annotated genes within each module. The gene ontology (GO) terms for biological processes are indicated together with enrichment score (E) of the process and Fisher *P* value

The module saddlebrown that tended to be correlated to ADFI and %backfat was composed of 16 unique genes participating to the response to stimulus. The skyblue module that was significantly correlated to muscle mass (%loin), showed a predominance of genes related to protein metabolism among its 44 unique constitutive genes (Table 2). The green module was a large module of 411 unique genes related to various processes such as epigenetics processes (chromatin organization, histone methylation), to cellular responses to compounds (nitrogen, organic cyclic) for stress and defense, and to circadian regulation. The brown was also a big module of 772 unique genes that regulated cell differentiation, protein and lipid processes, and signaling pathways.

### Hierarchy of expressed genes in the modules related to feed efficiency traits

To determine which expressed genes accounted the most in the correlations between the module eigengene (ME) and FCR, we calculated the Gene Significance (GS) in each module, and expressed the GS as a function of FCR. The top genes are listed in Table 3 and the all data are provided in Supplementary Table 3.

**Table 3:** Top genes in the molecular modules related to FCR

darkolivegreen	darkorange	darkred	lightcyan	royalblue	steelblue	violet	white
PTGER3	BMP6	SLCO2B1	ITGAD	POFUT1	UQCC2	SLPI	IGDCC3
TUBB6	PADI2	E4	DDC	LPAR3		P2RY1	TMEM14C
SLA-DRB1	DNAJB9	CCR3	HTRA1	CCR7		MUC4	KIAA0247
	WBSCR27	SLC46A2	EBF1	PTTG1		MED8	HRH1
	TAOK3	EIF1B	CBR3	NIPSNAP3B		HSPA1B	HTR7
	SERHL2	ZFAND6	MYBL1	EPHB6		TR10D	AFF1
		CD300C	C4BPB	SLC4A11		HSP70.2	ZUFSP
		FAM102A	KIAA0556	STRN			LDB3
		RPL14	MS4A1	ZCCHC10			PROX1
		WWP1	SLA-DOA	FMNL3			ACVR2B
		KRTCAP3	HMG20A	SKAP1			DGKA
		POLB	CYAC3	NPY			LUM
		PIGL	RALGPS2	PPP1R26			TBC1D19
		TMEM52B	ELL3	IZUMO4			
		PLA2G12A	PIKFYVE	NIPSNAP3A			

The unique genes corresponding to the annotated probes were listed in each module according to their GS.FCR value. In the table, only genes with a value greater than 0.3 for GS.FCR are indicated. The GS.FCR is the correlation between Gene Significance (GS) of the module eigengene and FCR

**Fig. 3:**
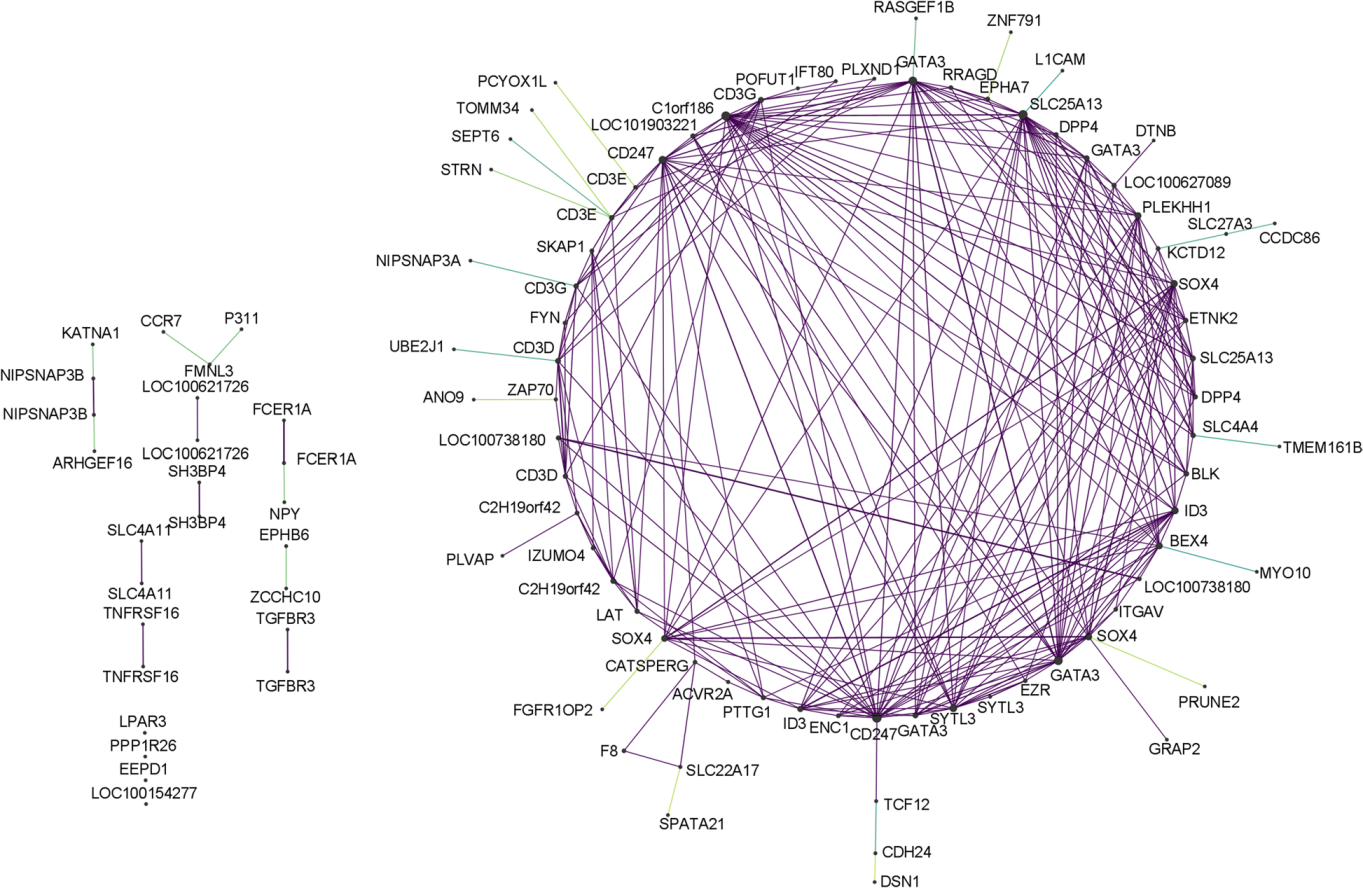
Graphical representation of the royalbue module. The network was constructed from the adjacency matrix of the royalblue module using Cytoscape. The nodes are the molecular probes, labeled with their annotation and connected by purple edges that represent the correlation coefficients greater than the 95*th* percentile and by green edges, for probes that were not sufficiently correlated to other probes, to the annotated probe to which they were the most correlated. The size of the nodes is a function of their degree

For the white module, IGDCC3, TMEM14C, HRH1, HTR7 and AFF1 were notably found as top genes. For the violet module, SLPI, P2RY1, MUCA, MED8, HSPA1B and HSP70.2 were the most important genes triggering the correlation of the module with FCR. For the royalblue module, POFUT1, LPAR3, CCR7, PTTG1, STRN, NPY and PPAR26 were pointed as important in the correlation with FCR. For the darkred module, EIF1B, RPL14 and KRTCAP3 were among the top 15 genes. For the lightcyan module, ITGAD, DDG, HTRA1, EBF1 and CBR3 were notably listed. A graphical representation of the importance of the genes in the modules is also provided. For the royalblue (Fig. 3), this shows that the probes that were highly correlated to other probes were listed in the top list of probes based on their module membership (MM).

### Metabolic profiles in the whole blood

The second part of the present study addressed the metabolic level by considering several variables obtained in the circulating blood of the 47 pigs, after non-target analysis (1H-NMR spectra) of plasma and specific analysis of the lipidic fraction (gas chromatography-derived information) of plasma.

**Fig. 4:**
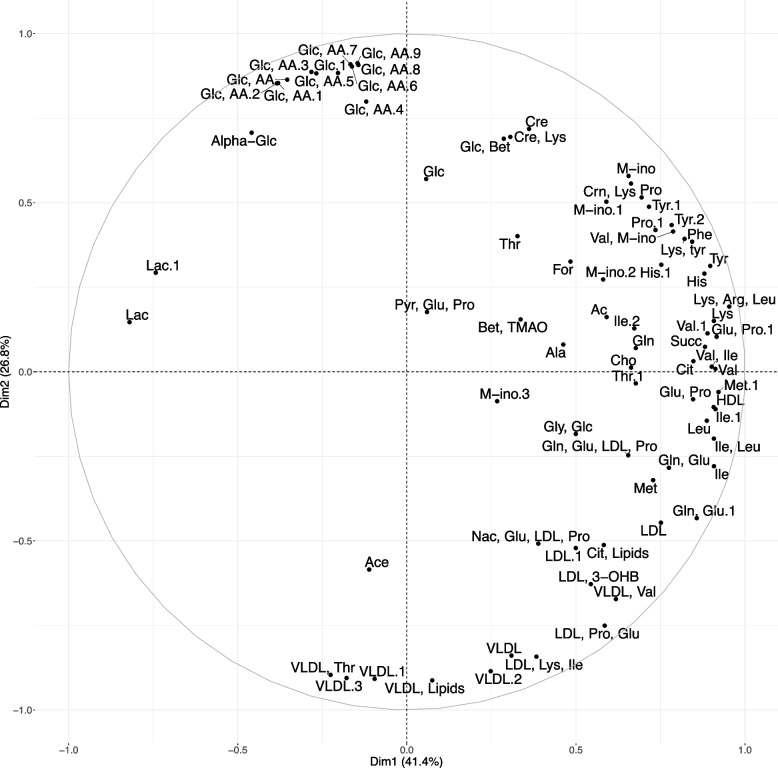
Correlation circle of the principal component analysis summarizing the profiles of circulating metabolites. 1H-NMR spectra were obtained in the plasma prepared from the whole blood of 47 growing pigs. The matrix of correlations was calculated from 94 individual variables corresponding to the different annotated spectra

The relationships between the 94 variables obtained from the 1H-NMR spectra were summarized by the first five dimensions of a principal component analysis (PCA). We showed that the two first dimensions represented $41.4\%$ and $26.8\%$ of the total variability, respectively. Figure 4 shows the corresponding correlation circle. The first dimension of this PCA mainly opposed lactate to the majority of the identified amino acids (lysine, tyrosine, valine, phenylalanine, methionine, leucine, isoleucine, glutamine-glutamate) and to high density lipoproteins (HDL). The second dimension mainly opposed glucose, eventually combined with other molecules, to circulating lipoproteins (very low density lipoproteins VLDL, low density lipoprotein LDL, and lipids) and to threonine. The third, fourth and fifth dimensions represented respectively $7.9\%$, $6.0\%$ and $3.8\%$ of the total variability. The third dimension mainly opposed circulating concentrations of glutamine (Gln), glutamate (Glu) and proline (Pro) on one hand, and beta-hydroxybutyrate on the other hand. The fifth dimension opposed circulating concentrations of betaine (bet) and trimethylamine N-oxide (TMAO; i.e., a metabolite produced by the liver and associated with microbiote metabolism), to VLDL and inositol concentrations. Correlation circles for the third, fourth and fifth are available in Supplementary Figs. 1 and 2.

**Fig. 5:**
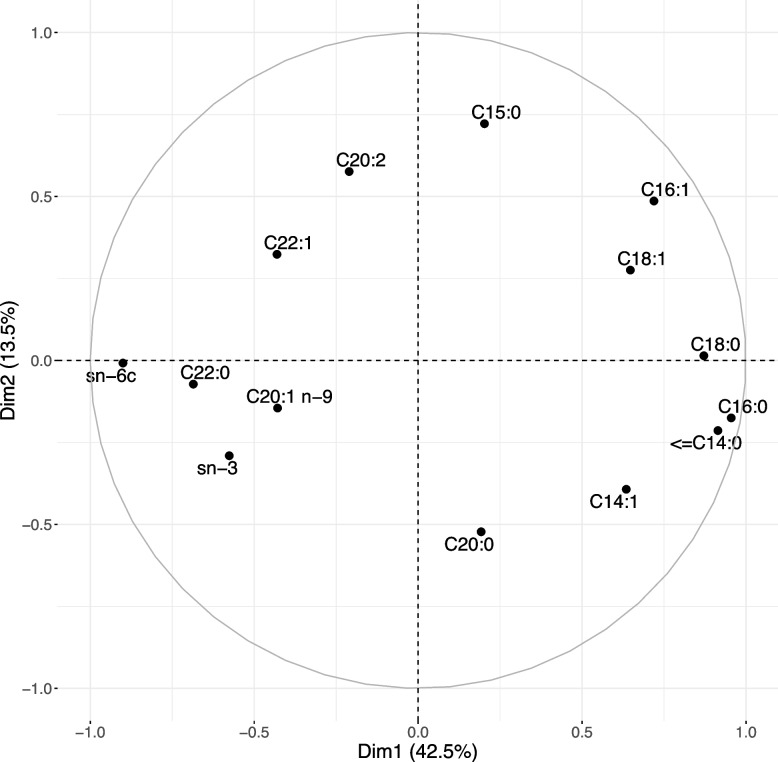
Correlation circle of the principal component analysis summarizing fatty acid composition in blood. The fatty acid composition (in percentage) was obtained in the plasma prepared from the whole blood of 47 growing pigs by using gas chromatography. Some of the individual FA were grouped in biologically relevant families (saturated FA with 14 carbons or less, omega-6 sum of $n-6$ and omega-3 sum of $n-3$), whereas the other fatty acids were kept as these

Considering specifically the lipid fraction of the plasma, we analyzed the fatty acids (FA) composition by target methodology. From the 30 FA (10 to 22 carbon chains) that can be analyzed, some of them were present in negligible concentrations in the plasma or even cannot be detected from the background in some pigs (e.g.; C10:0, C12:0, C18:4 n-3). Therefore, parts of the FA were grouped in biologically relevant families (saturated FA with 14 carbons or less, n-6 FA family; n-3 FA family). This led to a total of 14 variables representing single FA or groups of FA. They were then represented by a second PCA to summarize the profiles in circulating FA among the 47 pigs. Figure 5 shows the corresponding correlation circle. The first dimension represented $42.5\%$ of the total variability and opposed omega-6 (n-6) family of FA and to a lesser extent omega-3 (n-3) FA, to saturated family of FA. The second dimension represented $13.5\%$ of the total variability and opposed C15:0 to C20:0 FA. The third, fourth and fifth dimension represented respectively $8.0\%$, $7.5\%$ and $7.1\%$ of the total variability. The third dimension opposed C20:1 to C20:2 FA on one hand and C20:0 FA on the other hand, whereas the fourth dimension opposed the sum of n-3 FA to C20:1 FA. Correlation circles for the third, fourth and fifth are available in Supplementary Figs. 3 and 4.

### Connecting the two omics levels

We calculated the correlations between the eigengenes of the molecular modules (ME) and the profiles in circulating metabolites or fatty acids represented by the different dimensions of each PCA. This allows connecting the transcriptome (*via* the WGCNA modules) and the metabolome (*via* the principal components of the PCA).

The numbers of modules for which ME was correlated with at least one of the five dimensions of each PCA are presented in Table 4. A heatmap representing the correlation coefficients calculated between ME of all modules identified from microarrray data and the first five dimensions of the PCA, is available in Supplementary Figs. 5 and 6.

**Table 4:** Relationships between transcriptomic and metabolomic levels

	Dimensions of the principal component analysis of the metabolic level in blood					Dimensions of the principal component analysis of the fatty acids in blood				
Modules	Dim1_met	Dim2_met	Dim3_met	Dim4_met	Dim5_met	Dim1_FA	Dim2_FA	Dim3_FA	Dim4_FA	Dim5_FA
Significantly correlated ($P value \le 0.05$)	1	2	8	0	11	2	1	1	1	1
Trend ($P value \le 0.1$)	3	5	6	7	6	4	0	3	3	0

Modules of co-expressed genes were identified from microarray data in the whole blood by using weighted gene correlation network analysis (WGCNA). Circulating biochemical molecules in the plasma were analysed by 1H-NMR (metabolites; met) or target gas chromatography for the lipid fraction subset (FA) and the data were summarized by weighted linear regressions (dim) using principal component analysis (PCA). Correlations were calculated between the module eigengenes (ME) and the first five dimensions of each PCA. The table indicates the number of significant correlations or trends between modules of coexpressed genes and PCA dimensions

**Fig. 6:**
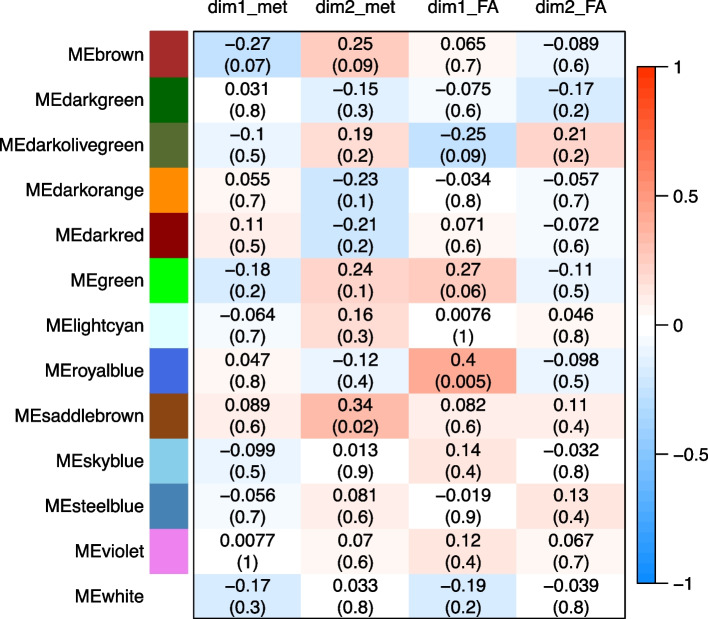
Heatmap of correlations between molecular modules and profiles of circulating molecules. Modules of co-expressed probes were obtained from a weighted gene correlation network analysis (WGCNA) from microarray data in the whole blood of 47 growing pigs. The eigengene of each module (ME) was considered as a mathematical representative of the expression levels of the molecular probes within the module. Circulating biochemical molecules were analyzed and the data were summarized by weighted linear correlation using principal component analysis (PCA). The first two dimensions of the PCA were called dim1_met and dim2_met for the metabolites obtained by 1H-NMR high throughput method and dim1_FA and dim2_FA for the fatty acids analyzed by target gas chromatography, respectively

There were few associations between molecular modules and metabolic and lipid profiles. A summary is presented in Fig. 6 considering only the list of modules of interconnected genes that have been found to be associated with the phenotypic traits of interest in the previous Definition of gene co-expression network in the whole blood of pigs section. There were no significant correlations between the eigengenes of the modules associated with FCR and the profiles in circulating metabolites. Only the darkorange module tended to be associated with the second dimension (dim2_met) which opposed circulating concentrations of glucose to circulating concentrations of LDL and VLDL lipoproteins. For the other animal traits, the darkgreen module which was significantly associated with ADFI, was highly correlated with dim3, which summarized the circulating concentrations in some amino acids (Gln, Glu and Pro) and hydroxybutyrate. The saddlebrown module which tended to be associated with ADFI and %backfat, was significantly correlated to the second dimension (dim2_met), and also tended to be associated with the fifth dimension (dim5_met) which opposed betaine and TMAO circulating concentrations to inositol concentration. Interestingly, more correlations were generally observed between dim*i*_met and modules related to %loin: the darkgreen module was highly correlated with dim3_met, and the brown module was correlated with dim3, and to a lesser extent with dim1_met, dim4_met and dim5_met.

Regarding plasma concentrations of FAs, we observed that the module eigengene (ME) of the royalblue module, that was negatively correlated with FCR and positively correlated with ADG, was highly positively correlated to the first dimension (dim1_FA) of the PCA. The darkorange module that was also negatively correlated with FCR, tended to be correlated with the fourth dimension (dim4_FA). The ME of the green also tended to be positively correlated with the first dimension (dim1_FA), whereas ME of the darkolivegreen tended to be negatively correlated.

## Discussion

### Analyzing inter-individual variability in feed efficiency

In animal and plant breeding, there has been an increasing interest in intermediate omics traits such as metabolomics and transcriptomics that mediate the effect of genetics on the phenotype of interest [21]. This study confirms that analyzing transcriptome in the whole blood and metabolome in plasma of growing pigs enables to depict the biological molecular pathways involved in various phenotypic traits related to feed efficiency (FE), namely feed conversion ratio (FCR) (i.e., the on-farm measure of feed efficiency) and average daily gain (ADG) (i.e., one of the component of FCR describing growth rate during the test period), and to a lesser extent, body composition (%loin, %backfat). This study is a step ahead for the understanding of the relationships between entities that can act in the inter-individual variability in these traits. Indeed, previous studies have rather addressed differentially-expressed (DE) genes [7, 8], gene set enriched pathways [22] or metabolic signatures [5, 14, 23] between the lowest and the highest FE animals in pigs, cattle or sheep. They have thus compared a gene or a molecule with itself in different conditions such as the response to selection for RFI (a measure of net feed efficiency) or extreme groups based on RFI or FCR. This does not enlighten and explain the interactions between entities in the architecture of the traits, and the behavior of regulatory genes acting in complex traits [24]. Moreover, the aforementioned studies often analyzed only a single type of omics data. Even when they included serum biochemistry in addition to transcriptomics [25] to illustrate consequences of variations in gene expression profiles, they did not intent to depict the correlations between the two levels of life organization (i.e., gene expression profiles and metabolites). The present study used networks approaches to reveal the main biological processes that are associated with the inter-individual differences in animal traits related to FE. Networks approaches are based on the assumption that the effect of the change in the expression level of one entity can be propagated through the interactions on other entities to orchestrate complex phenotypes [26]. We identified a total of 33 sub-networks (modules) of co-expressed genes in the whole blood across 47 pigs. The network was built with a low threshold set for the minimum number of co-expressed entities in the modules, with the assumption that this may facilitate not only the identification of co-expressed but also of co-regulated genes. Finding one to three clusters of enriched biological processes for the majority of the molecular modules argues for the homogeneity in biological processes shared by the co-expressed genes participating to each module. Furthermore, modules in close-vicinity in the dendrogram (hierarchical clustering), such as the darkolivegreen, lightcyan and steelblue modules or the royalblue, darkred and violet modules, respectively, did not share identical GO terms. This argues for keeping these modules separated rather than merging them. Altogether, the procedure used for network building in this study was then adequate for the identification of co-regulated entities in the different modules.

### Enriched pathways in co-expressed genes modules related to variability in feed efficiency

From the 33 modules identified in the whole genes network across the 47 pigs, six modules were significantly associated with FCR and two modules tended to be related to FCR. In the whole blood of young pigs, another study [8] has previously identified four co-expression modules (minimum of 30 genes per module as threshold, leading to 89 to 786 genes per modules) in the low or high RFI groups, and indicated that DE genes overlapped with each of the four differentially expressed modules; however, they found only one module that was significantly correlated to the RFI phenotype. Moreover, 34 modules of co-expressed genes were identified [24] from RNA-seq analysis in the liver of low vs high RFI cattle (using a threshold of 30 genes as the minimum in each module), out of which four modules showed significant correlations to RFI. Importantly, the majority of the modules related to FCR in the present study were also related to ADG and to %loin, whereas none of them were significantly associated with individual feed intake (ADFI). This suggests that the main molecular entities in the whole blood explaining the inter-individual variations in FE were involved in the determinism of lean growth potential rather than acting in the regulation of feeding behaviour. The pigs used in this study originated from a divergent selection for RFI and fed different diets during the test period. Because ADG is an independent variable in the regression that estimates predicted feed intake, RFI and ADG have no correlation. The situation is however quite different for FCR, since ADG is part of the ratio in the calculation. Although Gilbert and colleagues [2] indicated low responses (although statistically significant) to RFI selection on lean meat content across generations of pigs, several studies have consistently found a higher proportion of lean pieces in the most feed efficient pigs as induced by genetic selection [27, 28] or by management strategies [29]. Skeletal muscle is the largest organ in the body and plays important roles in the utilisation and storage of a large proportion of the energy from feed. This likely explains why the molecular modules related to FCR were also partly associated with %loin.

To depict the biological functions of the modules identified in the current study, a functional enrichment analysis was performed within the modules separately. Five of the aforementioned eight modules related to FCR, the violet, lightcyan, darkorange, royalblue and darkolivegreen, were significantly enriched in immune and defense-related processes. In the whole blood, immunity and stress response have been previously identified as biological pathways shared by DE genes between low and high efficient pigs [8]; however, correlation analysis to RFI phenotype rather suggested the importance of a module of co-expressed genes participating to cell adhesion, apoptotic process and immunoglobulin production. In the present study, the importance of immunity and defense-related pathways in the architecture of FCR trait may be over-estimated, since we considered the blood where these processes are specifically enriched. However, previous studies on DE genes in pigs have also reported differences in expression levels of genes involved in defense pathways when examined in different tissues (liver, skeletal muscle, adipose tissue) between divergent lines for RFI [28]. In cattle, [25] also found an enrichment of the transcriptomic networks in the inflammatory response, regulation of monocyte differentiation, proliferation and differentiation of T lymphocytes in the liver from animals with low or high RFI. Since the whole blood reflects the concerted actions of the different tissues, these data support the current findings that immunity and defense-related pathways are important in the determination of feed efficiency. Finally, the recent identification of variations in expression of genes associated with the immune system in milk from low vs high FE dairy sheep [30] , further argues for the informative potential of immune and defense pathways to depict feed efficiency phenotypes of farm animals when analyzed in various biological fluids. Defense mechanisms trigger the use of nutrients for basal metabolism rather than for production performance. This likely explain the importance of defense mechanisms in the determination of feed efficiency, through regulations of ADG and %loin. In support, [31] shows that high RFI piglets (the less efficient animals) had greater resting energy expenditure and respiratory quotient than low RFI piglets (the most efficient). Among defense mechanisms, we suggested that T cell signaling (royalblue) was negatively related to FCR, whereas B cell activation (lightcyan) and inflammation (darkolivegreen) were positively associated to FCR. This association is likely due to the fact that inflammatory stimulation was associated with a re-orientation of nutrients and alteration of metabolism [31] in growing pigs, thus deteriorating feed efficiency of the animals.

In the present study, other biological processes were also enlightened as important contributors to the inter-individual variability of FCR. Indeed, three modules were composed of co-expressed genes involved in translation (darkred), protein localization (steelblue) or circulatory system development and learning (white). In the whole blood of cattle, a set of genes associated with the metabolism of proteins was also identified as the most enriched pathway of genes differentially inhibited or activated in high-RFI when compared to low-RFI beefs [22]. These pathways could more specifically account for the regulation of lean growth rate, because significant correlations with ADG and %loin were also identified. Finding nitrogen metabolic process (skyblue), protein modification and muscle cell differentiation (brown) as enriched processes in modules related to %loin further support the assumption that whole blood can encompass molecular mechanisms involved in muscle development and metabolism. Finally, two modules, the darkgreen and saddlebrown, were or tended to be related to both ADFI and %backfat, a surrogate of body adiposity. The darkgreen module encompassed genes involved in the response to toxic substances. In accordance, there is generally a marked reduction in voluntary feed intake in disease-challenged pigs [32].

### Important genes in molecular networks related to feed efficiency

The main objective of the current study was to enlighten interaction networks related to feed efficiency, rather than focusing on single genes. However, when looking at the hierarchy of the genes in the molecular modules found as underlying FCR, we pointed RPL14 in the darkred module. This gene has been previously suggested by bioinformatics as a hub node gene in regulatory networks [33]. This is an important point to argue for the biological relevance of gene network architecture built herein. Moreover, some of the genes contributing the most to the association between transcriptomics level and animal trait as ranked according to (GS.FCR) values in each module, have been previously identified as genes with fold changes in their expression level greater than |2| between groups of low and high (RFI) pigs [7]. There were SLPI (violet module), EIF1B (darkred module) and HTRA1 (ligthcyan module). As expected, these genes are participating to different biological processes listed as specifically enriched in their parent modules, such as immune response by protecting epithelial surfaces (SLPI), regulation of cell growth (HTRA1) and translational initiation (EIF1B). Of note, HTRA1 encodes a secreted enzyme that may regulate the availability of insulin-like growth factors (IGFs), and correlated responses of IGF-I to RFI have been observed in pigs [34]. Altogether, these genes are likely biologically important in the variability of FCR.

The royalblue module is of upmost interest since it was associated with FCR and with profiles of circulating fatty acids (see next section). Therefore, the molecular functions of its top-ranked genes deserve deeper investigations in the Human Gene Database. The LPAR3 (Lysophosphatidic Acid Receptor 3) and CCR7 (C-C Motif Chemokine Receptor 7) genes are members of the G protein-coupled receptor family (GPCR). Especially, the protein encoded by CCR7 is known to activate B and T lymphocytes and to control the polarization of T cells in chronic inflammation. Another gene involved in T-cell signaling was SKAP1 (Src Kinase Associated Phosphoprotein 1) that is required for an optimal conjugation between T cells and antigen-presenting cells. Although EPHB6 (EPH Receptor B6) codes for a protein that mainly influences cell adhesion and migration and regulates cell developmental process rather than immunity, one of its related pathways is GPCR signaling. Interestingly, the GPCR pathway has been also identified as a putative candidate for RFI difference in pigs by genome-wide association studies [35]. Another member of the royalblue module is NPY, a gene coding for the neuropeptide Y that influences many physiological processes including stress response, food intake, energy balance and circadian rhythms. In accordance, hypothalamic genes expression including NPY plays a potential role in feed efficiency variation in different farm species [36]. The neuropeptide Y also functions through GPRC. Finally, among the top genes in the white module, HTR7 encodes the serotonin receptor which belongs to the GPCR family and is regulating several behaviours of animals.

### Relationships between transcriptomic and metabolic levels in the definition of feed efficiency or related traits

Combined phenotype-metabolome-genome analysis by inferring gene networks based on partial correlation and information theory approaches has been valuable to confirm cellular maintenance processes as major contributors to genetic variability in bovine feed efficiency [37]. Therefore, the present study also addressed the interactions between two levels of organization in the circulating entities, i.e., transcriptome and metabolome, and their relations to productive traits in the pigs. Variations at the metabolic level were first summarized by linearly transforming the data into few new coordinates that explained most of the total variance, thanks to principal component analysis (PCA). Correlating the PCA coordinates to the eigengenes of the 33 modules allows to determine whether pigs with the same profiles in circulating fatty acids (FA) and lipoproteins, amino acids (AA) or energy-related metabolites (glucose, lactate, betaine, etc.) shared similarities in groups of co-expressed genes. However, few significant correlations were identified between the two organization levels. The eigengene of the royalblue module was highly correlated to the PCA coordinate that associated circulating concentrations in saturated FA and in polyunsaturated FA (omega-6 FA, and to a lesser extent, omega-3 FA families). In other words, the greater expression levels of genes involved in T signaling, cell adhesion and cell developmental process in the whole blood, were associated with a higher proportion of saturated FA and a lower proportion of PUFAs in plasma, and altogether, these changes accounted for a better feed efficiency (lower FCR) and higher ADG). An important regulatory element underlying this association might be the expression level of LPAR3, a gene that is involved in phospholipid binding. Similarly, the eigengene of the darkorange module which was composed of co-expressed genes related to immune process, tended to be correlated with the circulating concentrations of omega-3 FA, and these processes simultaneously accounted to FCR. Among the top-ranked genes in this module, BMP6 encodes a secreted ligand of the transforming growth factor (TGF-beta) superfamily of proteins that regulate a wide range of biological processes including fat cell development, and TAOK3 encodes a serine/threonine protein kinase that activates the p38/MAPK14 stress-activated MAPK cascade, a pathway regulating also adipose cell development and metabolism. Thus, whereas the gene network approach did not identify any enriched pathways related to lipid metabolism in the whole blood transcriptome, the combination between transcriptomics and metabolic data suggests that fatty acid metabolism in different tissues can be related to FCR and further influenced/be influenced by interconnected molecular pathways of genes related to immunity. In support, muscle of high-FE pigs exhibited lower proportion of saturated FA and an enhanced proportion of polyunsaturated FA when compared with low-FE pigs [27], and co-expression analysis in the liver has revealed altered lipid metabolism between high and low feed efficient steers [25]. Relationships between FA metabolism and immunity have been also described in the literature, showing that omega-3 (n-3) PUFA can suppress T cell antigen presentation, activation, proliferation and cytokine expression [38]. In addition, high fat western diet promotes inflammation and modifies immunity [39].

Among significant associations identified between modules of co-expressed genes in the whole blood and profiles in circulating metabolites, the darkgreen module composed of genes involved in the responses to lipopolysaccharide (LPS) and toxic substances and related to ADFI, was associated with the circulating concentrations of beta-hydroxybutyrate and of Pro, Glu and Gln amino acids. Beta-hydroxybutyrate is a ketone body whose concentration is rising up after fasting and in situation of energy deficit, illustrating its dependence to the regulation of feed intake. Sensing of AA can also act on the hypothalamic control of food intake [40]. Networks of co-expressed genes related to %loin (such as brown and green) also displayed significant correlations with the metabolome profile, which suggests strong relationships between AA metabolism and the molecular regulation of muscle growth in the pigs. In support, protein (amino acids) metabolism is essential for optimizing efficiency of nutrient absorption and metabolism and to enhance growth performance. Relationships between transcriptomics and metabolomic data to depict the biological processes underlying complex production traits like FCR, ADG, ADFI, have been identified herein by statistical analyses (multivariate-based procedures for data concatenation) and then, scrutinized with the functional annotation tools DAVID (pathway-based integration techniques) and expert knowledge about the potential roles of specific entities. However, when transcriptomic and metabolomic data are integrated, there is no direct association between metabolite and transcript. Although commercially available tools have been developed to visualize ranked pathways among molecules, there are many biases when treating genes and metabolites as equivalent entities [41]. To explore the causality within the interconnected entities at the different levels of cell organization, it seems necessary to use the graph theories. First, the feed efficiency networks identified herein could be compared in their topologies (direct interactions, connectivity degree per gene, etc.) with random networks [37]. Second, knowledge graphs can be generated thanks to web semantic-dedicated queries to identify paths composed of chains of relationships. Path lengths between entities (pairs of co-expressed genes and small molecules), traversed properties (edges) and encountered biochemical reactions could be then analyzed. However, the mapping between different identifiers of genes/metabolites in naming systems is still a problem to be overhelmed in this process [41].

In conclusion, the inter-individual differences in feed conversion ratio (FCR, i.e., the on-farm measure of feed efficiency), were inferred to be mainly due to variation of co-expressed genes participating to immunity, defense mechanisms, inflammatory response, cell developmental process, translation and protein localization. These variations induced changes in the capacity of amino acids usage and lipid (fatty acids) metabolism between pigs. Among the component traits of FCR, these processes accounted likely more in the variation of growth rate than in the regulation of feed intake. However, few genes in the gene networks (e.g., NPY) are suggested for their roles in regulating feeding behaviour. Analyzing the gene network also allowed to propose integrative regulatory mechanisms such as G protein-coupled receptors (GPCR). Relationships were indicated between T cell receptor signaling, cell development process and circulating concentrations of omega-3 fatty acids in plasma, which both underlined inter-individual variability in feed efficiency. This suggests that nutritional recommendations for growing pigs should consider the lipid fraction of diets to improve health and production traits in synergy.

### Supplementary Information

**Additional file 1.**

## Data Availability

Transcriptomic dataset was obtained from NCBI’s Gene Expression Omnibus (GEO) Subserie accession number GSE70838 (http://www.ncbi.nlm.nih.gov/geo/query/acc.cgi?acc=GSE70838). The metabolomic dataset was retrieved in Jegou et al. [14]. Phenotypic traits and fatty acid composition in plasma are are deposited in the publicly available repository at https://entrepot.recherche.data.gouv.fr/dataset.xhtml?persistentId=doi:10.57745/TM2ANC. The adjacency matrix is available at https://data-access.cesgo.org/index.php/s/YPz0J2ItxIEuN5M. We provide R notebooks detailing the analysis https://github.com/cjuigne/multiomics_and_feed_efficiency.

## Abbreviations

ADG: average daily gain
ADFI: average daily feed intake
DE: differentially-expressed
FCR: feed conversion ratio
GS: gene significance
ME: module eigengene
PCA: principal component analysis
