# Supplementary material for: Small networks of expressed genes in the whole blood and relationships to profiles in circulating metabolites provide insights in inter-individual variability of feed efficiency in growing pigs
Source: BMC Genomics. 2023 Oct 27;24:647. doi: 10.1186/s12864-023-09751-1 (PMC10605982; doi:10.1186/s12864-023-09751-1)
Supplement: Supplementary file 1 — Additional file 1. [file 12864_2023_9751_MOESM1_ESM.zip › additional files 1-2-4-5-6-7-8-9.pdf]

Additional file 1 — Table 1: Animal phenotypic traits and circulating fatty acids in the whole blood

Table 1: **Animal phenotypic traits and circulating fatty acids in the whole blood**.

**Phenotypic traits**		
	Mean	Standard Deviation
age at slaughter	132.7	3.70
weight at slaughter	75.69	7.44
ADG	840.2	111.20
ADFI	2280	212.64
FCR	2.738	0.25
%backfat	7.093	1.56
%loin	28.26	1.05
**Relative percentage of fatty acids**		
	Mean	Standard Deviation
C14:0	0.36	0.16
C14:1	0.31	0.23
C15:0	0.17	0.05
C16:0	14.81	3.1
C16:1	1.03	0.3
C18:0	12.56	2.19
C18:1	27.12	2.14
n-6	33.41	4.93
n-3	5.93	1.95
C20:0	0.15	0.24
C20:1	0.34	0.33
C20:2	0.84	0.75
C22:0	1.37	0.94
C22:1	1.42	0.8

A total of 47 growing pigs were considered during a test period of 58 days.
Abbreviations used: ADG = average daily gain; ADFI: average daily feed intake ; FCR = food conversion ratio; %backfat = weight of dorsal subcutaneous fat tissue (relative to carcass weight); %loin = weight of dorsal subcutaneous fat tissue (relative to carcass weight).

Additional file 2 — Table 2: Number of probes significantly linked to the animal phenotypic traits of interest (linear models).

Table 2: Number of probes significantly linked to the animal phenotypic traits of interest (linear models)

**Variable**	$p.value < 0.01$	$p.value < 0.05$
**line**	**1813**	**3726**
**age at slaugher**	**1174**	**4940**
diet	50	335
%backfat	164	726
ADFI	213	1352
ADG	325	1756
FCR	425	1682
%loin	359	1918
weight at slaugher	90	596

Additional file 3 — Table 3: Composition of the modules in probes with their annotation and GS.FCR and MM.
GS.FCR: Gene Significance for Feed Conversion Ratio (correlation between the FCR and each each probe), absGS: absolute value of the GS.FCR, MM: Module

Additional file 3.csv

Additional file 4 — Figure 1: Correlation circle of the third and fourth dimensions of the principal component analysis summarizing the profiles of circulating metabolites in the blood.

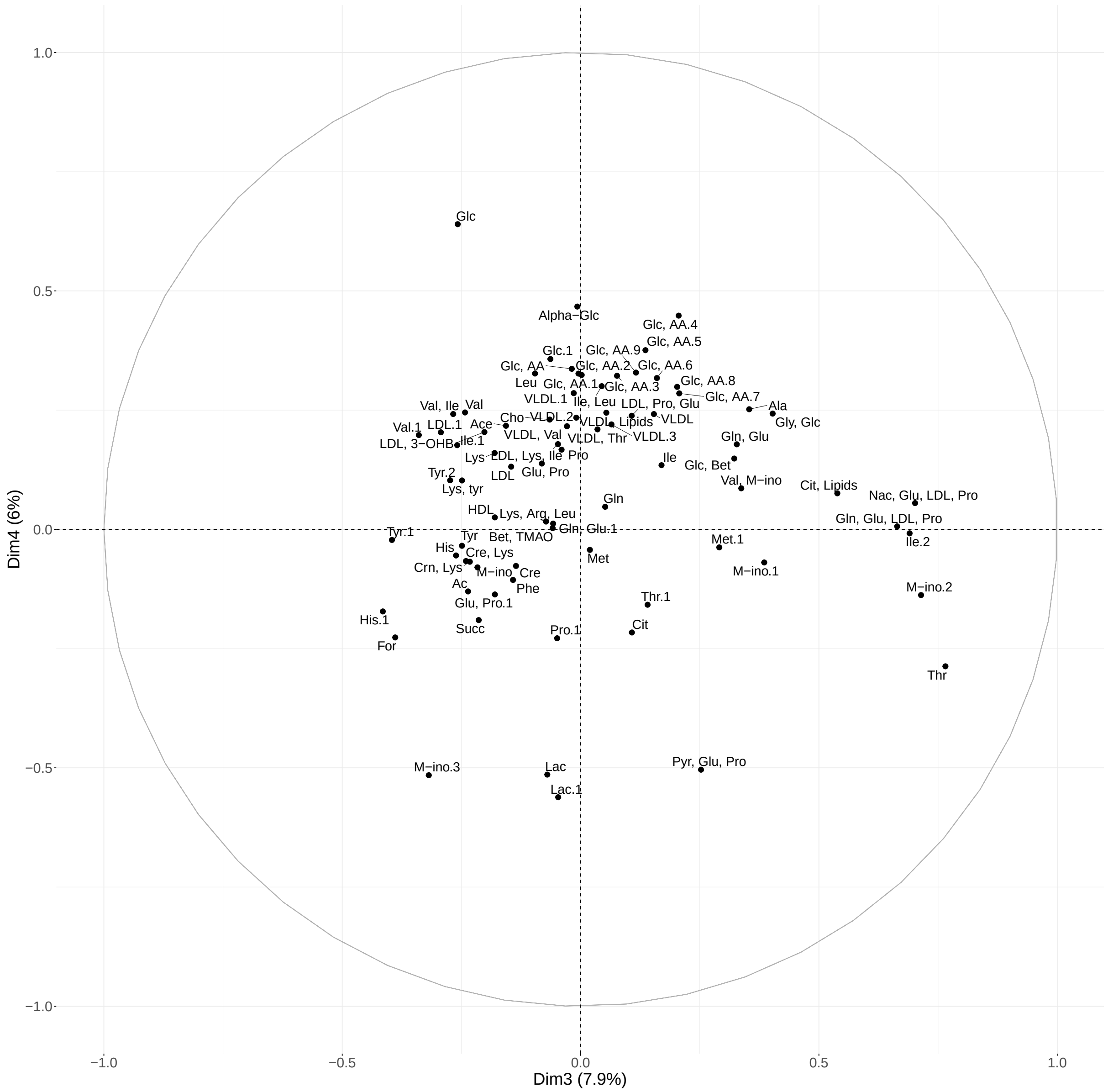

Figure 1: **Correlation circle of the third and fourth dimensions of the principal component analysis summarizing the profiles of circulating metabolites in the blood.**

1H-NMR spectra were obtained in the plasma prepared from the whole blood of 47 growing pigs. The matrix of correlations was calculated from 94 individual variables corresponding to the different annotated spectra.

Additional file 5 — Figure 2: Correlation circle of the third and fifth dimensions of the principal component analysis summarizing the profiles of circulating metabolites in the blood.

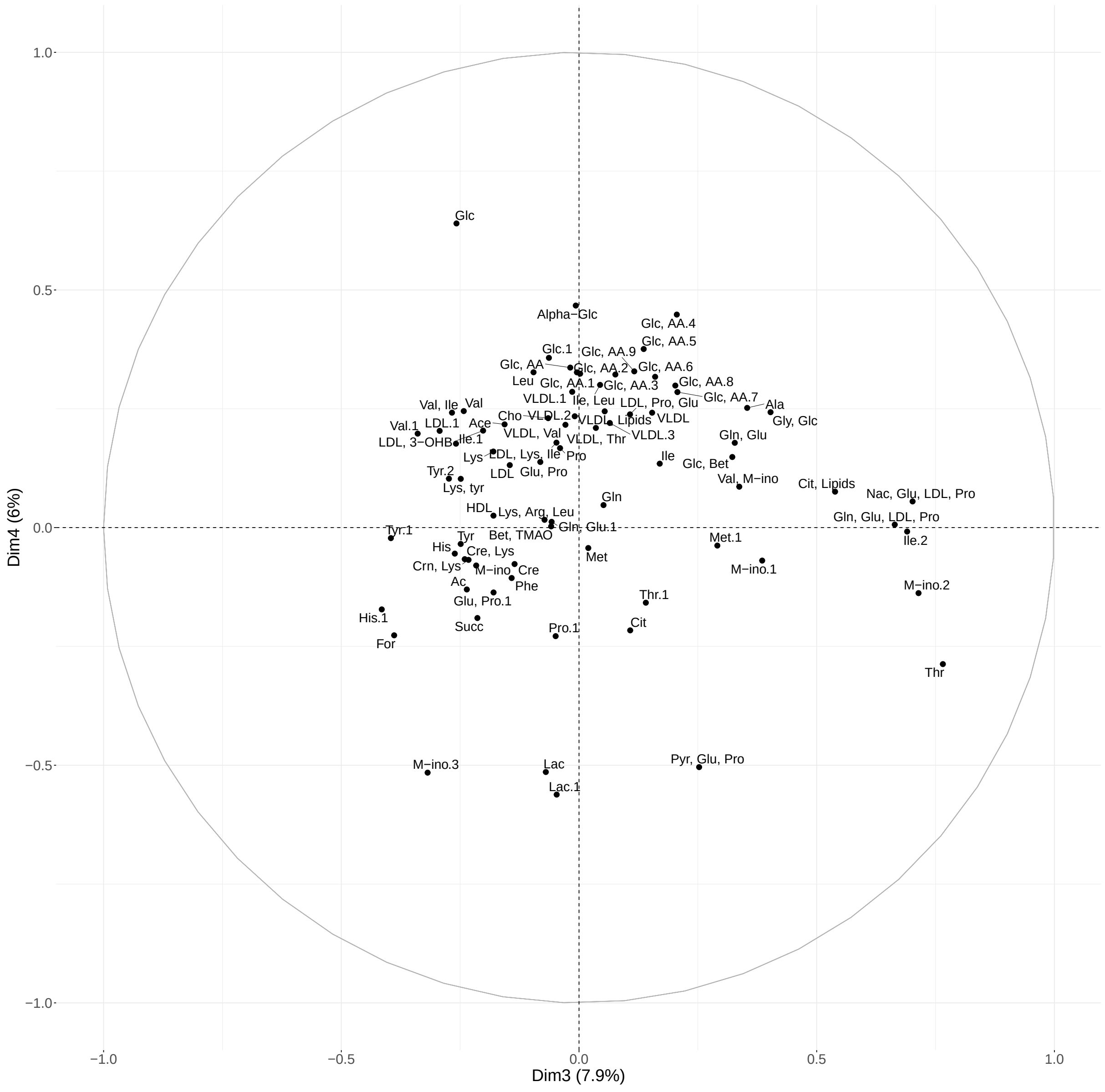

Figure 2: **Correlation circle of the third and fifth dimensions of the principal component analysis summarizing the profiles of circulating metabolites in the blood.**

1H-NMR spectra were obtained in the plasma prepared from the whole blood of 47 growing pigs. The matrix of correlations was calculated from 94 individual variables corresponding to the different annotated spectra.

Additional file 6 — Figure 3: Correlation circle of the third and fourth dimensions of the principal component analysis summarizing fatty acid composition in blood.

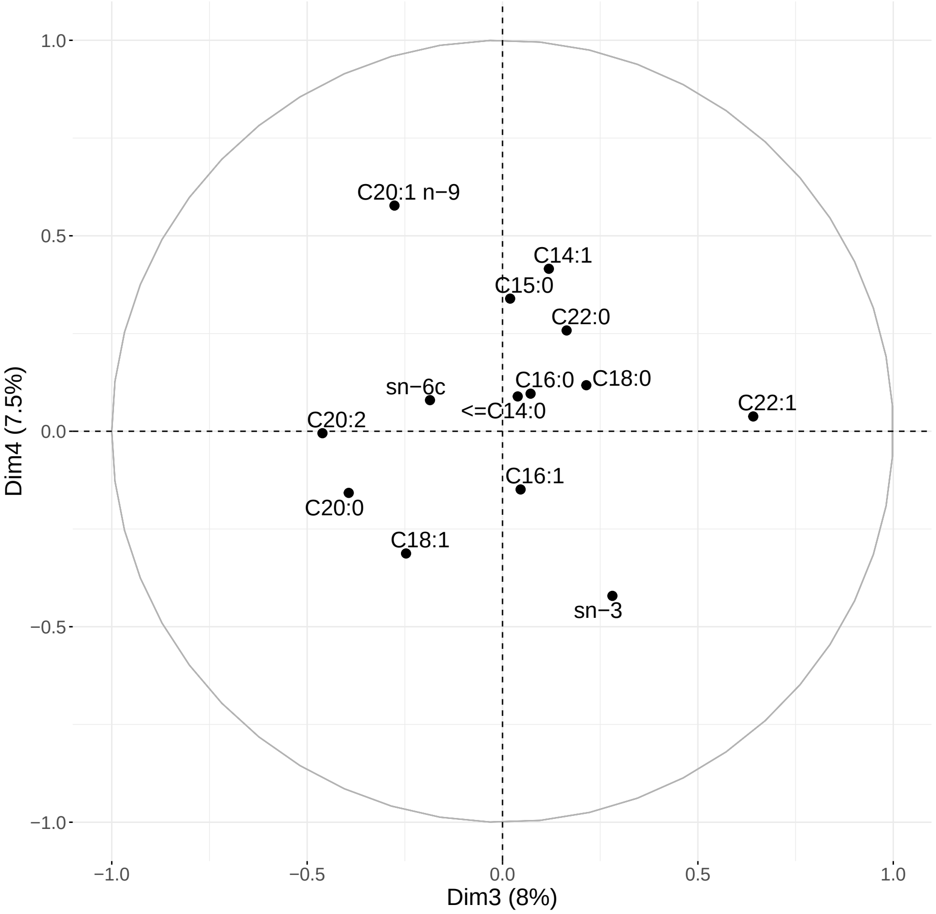

Figure 3: **Correlation circle of the third and fourth dimensions of the principal component analysis summarizing fatty acid composition in blood.**

The fatty acid composition (in percentage) was obtained in the plasma prepared from the whole blood of 47 growing pigs by using gas chromatography. Some of the individual FA were grouped in biologically relevant families (saturated FA with 14 carbons or less, omega-6 sum of $n-6$ and omega-3 sum of $n-3$), whereas the other fatty acids were kept as these.

Additional file 7 — Figure 4: Correlation circle of the third and fifth dimensions of the principal component analysis summarizing fatty acid composition in blood.

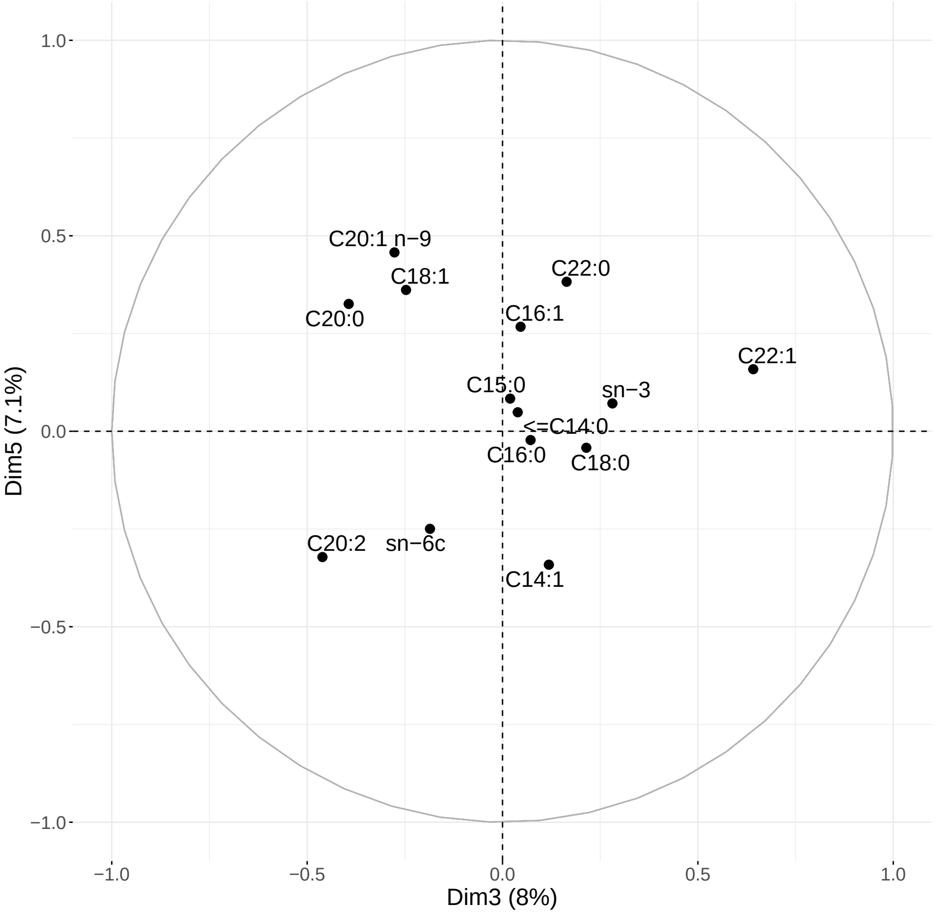

Figure 4: **Correlation circle of the third and fifth dimensions of the principal component analysis summarizing fatty acid composition in blood.**

The fatty acid composition (in percentage) was obtained in the plasma prepared from the whole blood of 47 growing pigs by using gas chromatography. Some of the individual FA were grouped in biologically relevant families (saturated FA with 14 carbons or less, omega-6 sum of $n-6$ and omega-3 sum of $n-3$), whereas the other fatty acids were kept as these.

Additional file 8 — Figure 5: Heatmap of correlations between module eigengenes of molecular modules of co-expressed genes in the whole blood and the first dimensions of principal component analyses summarizing the metabolites obtained by 1H-NMR high throughput method in plasma.

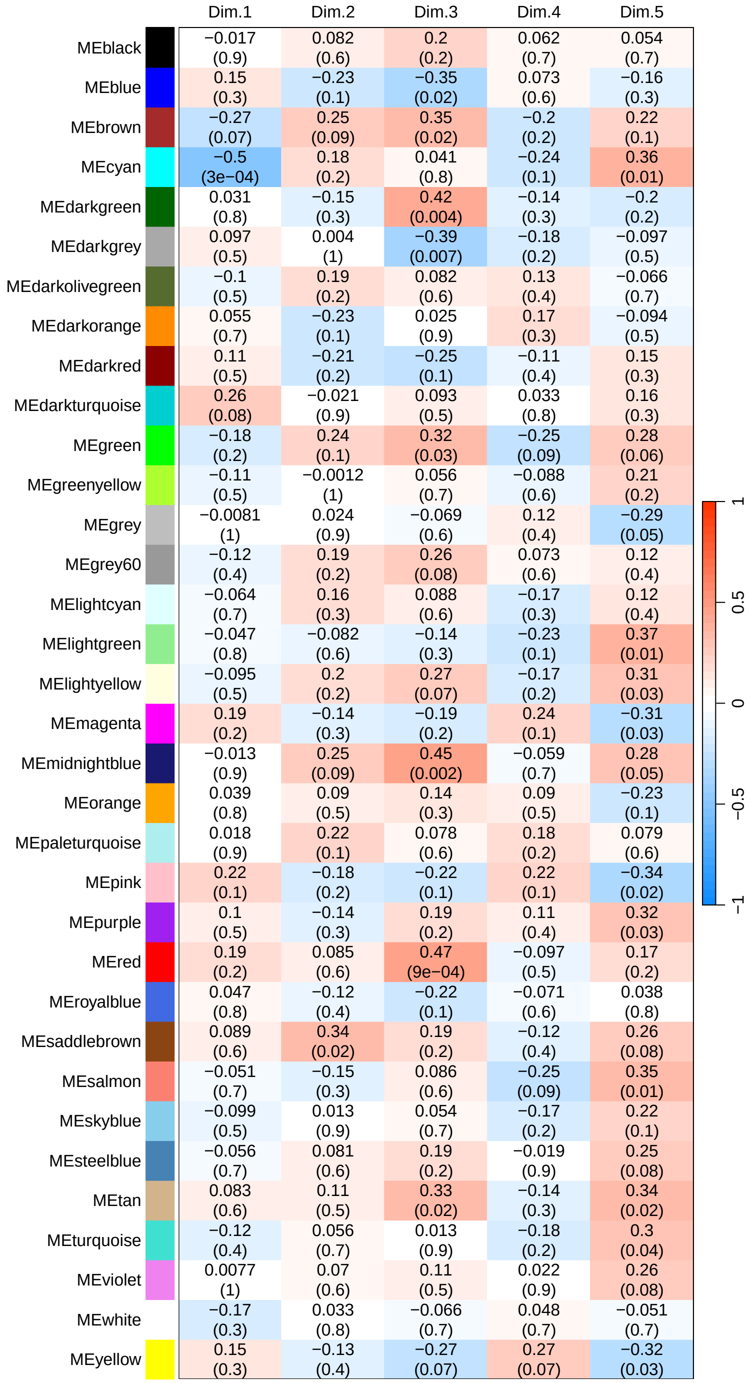

Figure 5: **Heatmap of correlations between module eigengenes of molecular modules of co-expressed genes in the whole blood and the first dimensions of principal component analyses summarizing the metabolites obtained by 1H-NMR high throughput method in plasma.**

Modules of co-expressed probes were obtained from a weighted gene correlation network analysis (WGCNA) from microarray data in the whole blood of 47 growing pigs. The eigengene of each module (ME) was considered as a mathematical representative of the expression levels of the molecular probes within the module. Circulating biochemical molecules were analyzed and the data were summarized by weighted linear correlation using principal component analysis (PCA). The ith dimensions of the PCA were called dim$i$_met.

Additional file 9 — Figure 6: Heatmap of correlations between module eigengenes of molecular modules of co-expressed genes in the whole blood and the five first dimensions of principal component analyses summarizing the fatty acids analyzed by target gas chromatography.

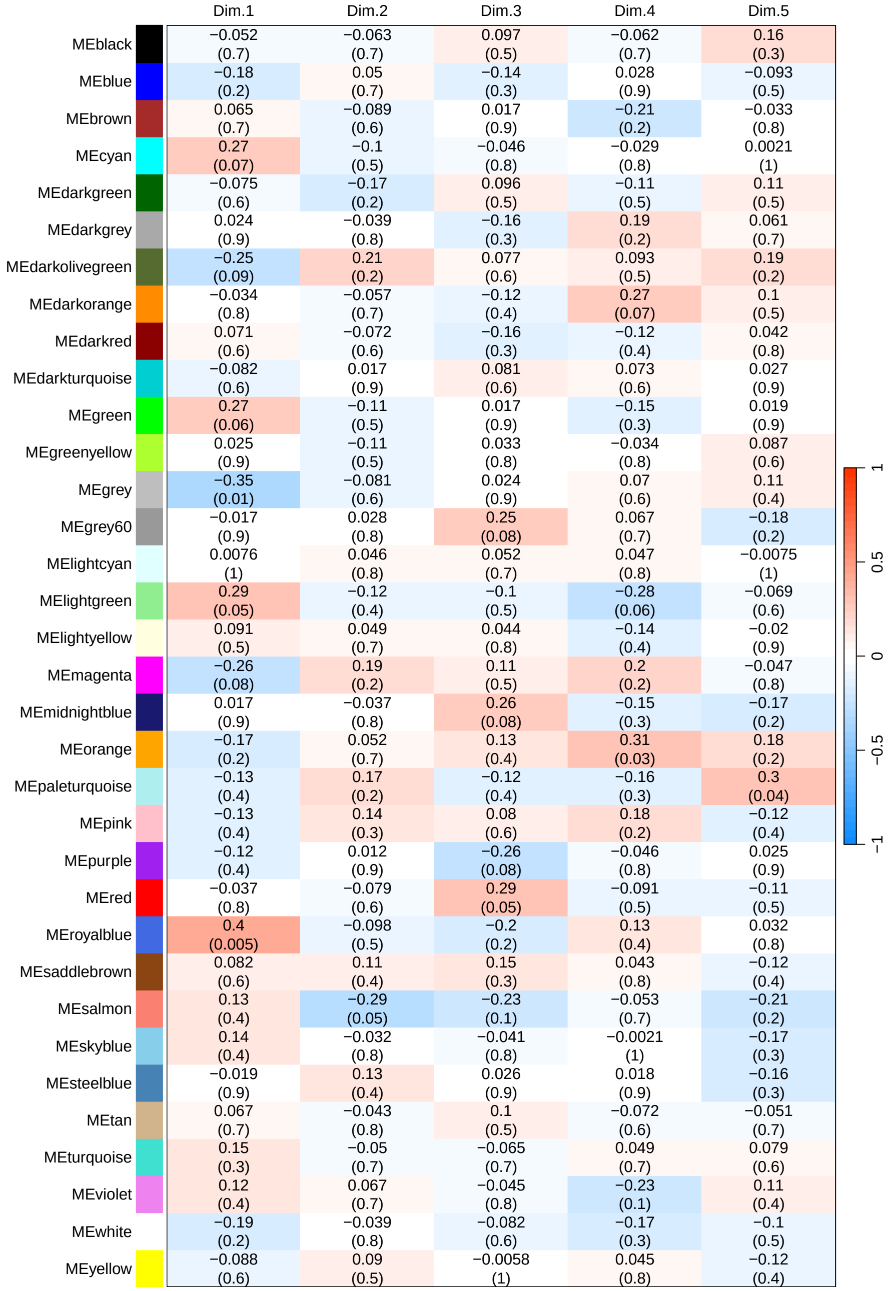

Figure 6: **Heatmap of correlations between module eigengenes of molecular modules of co-expressed genes in the whole blood and the five first dimensions of principal component analyses summarizing the fatty acids analyzed by target gas chromatography**.

Modules of co-expressed probes were obtained from a weighted gene correlation network analysis (WGCNA) from microarray data in the whole blood of 47 growing pigs. The eigengene of each module (ME) was considered as a mathematical representative of the expression levels of the molecular probes within the module. Circulating biochemical molecules were analyzed and the data were summarized by weighed linear correlation using principal component analysis (PCA). The ith dimensions of the PCA were called dim$i$_FA.
